# The long road to recognition: a historical review of amyloidosis from early pathology to disease-modifying therapy

**DOI:** 10.1007/s10741-026-10666-8

**Published:** 2026-08-26

**Authors:** Lauren N. Halverson, Brett W. Sperry

**Affiliations:** Saint Luke’s Mid America Heart Institute, Kansas City, 4401 Wornall Rd, Suite 2000, MO 64111 USA

**Keywords:** Amyloidosis, Light chain, Transthyretin, Heart failure, Neuropathy, Cardiomyopathy, Congo red

## Abstract

Within the last several decades, great strides are being made in both the diagnosis and treatment of cardiac amyloidosis, yet the condition itself has been a severely under-recognized cause of heart failure for centuries. For much of medical history, the accumulation of these insoluble fibril deposits was an enigmatic finding, observed primarily post-mortem in patients suffering from chronic inflammatory conditions. This review traces the fascinating evolution of amyloidosis: from its origins as a botanical term mistakenly applied to human pathology, through the serendipitous discoveries of histological stains like Congo red, to the molecular triumphs that identified the specific precursor proteins driving the AL, AA, and ATTR subtypes. By understanding the historical roadblocks and breakthroughs, we can better appreciate the modern diagnostic imaging and targeted therapies that are finally bringing this complex disease out of the shadows.

## Introduction

**Fig. 1:**
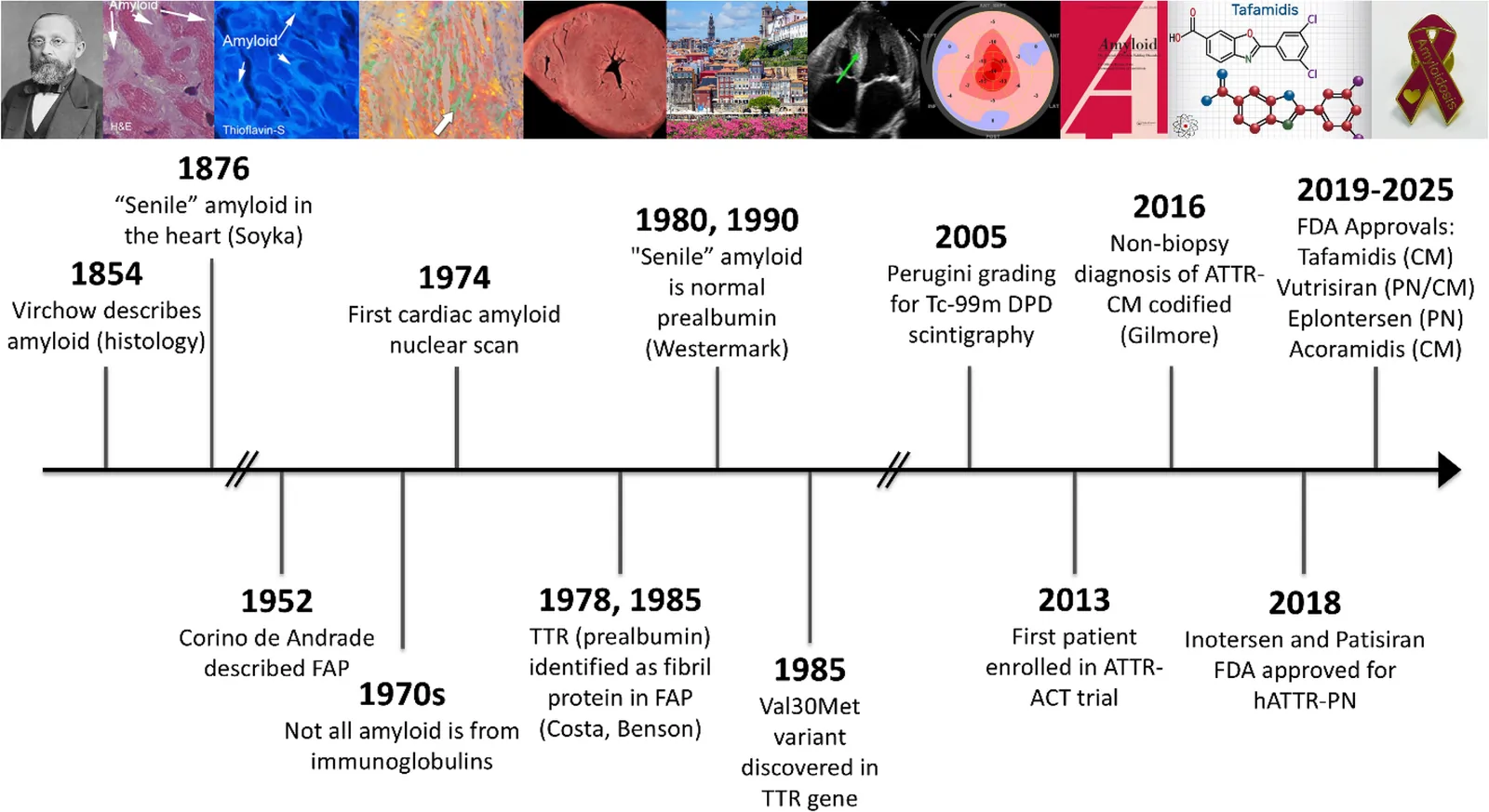
Timeline of Notable Discoveries in ATTR Amyloidosis. Developments in amyloidosis research spanning several centuries. While the first description of what was likely amyloid involvement of the spleen was in 1639 by Fontanus, Virchow first used the term ‘amyloid’ in 1854 [1, 2]. Subsequent clinical and pathologic work converged to identify the precursor TTR protein, clinical differentiation between familial and ‘senile’ ATTR amyloidosis, and differentiation from AL and AA amyloidosis. Recent advances in non-invasive cardiovascular imaging and treatment have led to a dramatic increase in the diagnosis rates of this condition

Before the advent of modern diagnostic tools, early scientists relied on gross pathology to diagnose amyloidosis, noting that affected organs took on an abnormal, fat-like appearance. These early anatomical observations, ranging from the “sago spleen” to the “lardaceous” liver, were typically seen in patients with chronic infections like tuberculosis or syphilis, representing what is now known as secondary AA amyloidosis. Scientists over the centuries have had various hypotheses for the pathophysiology, etiology, and treatment of this disease. Ironically, while modern science has since proven that none of these early researchers correctly identified the true composition of amyloid, many of the disease’s underlying causes remain enigmatic to this day. This historical review aims to trace the timeline of amyloidosis research (Fig. 1), exploring the transition from these early anatomical debates to the accidental discovery of vital staining methods to the modern molecular understanding of distinct amyloid protein structures. While recent reviews have focused largely on describing advancements in amyloidosis research, less attention has been given to the origins and subsequent progression of these developments. This review emphasizes the importance of understanding the historical evolution of amyloidosis to guide future innovation, serving as a complement to existing literature.

## Pathologic origins of amyloid

**Fig. 2:**
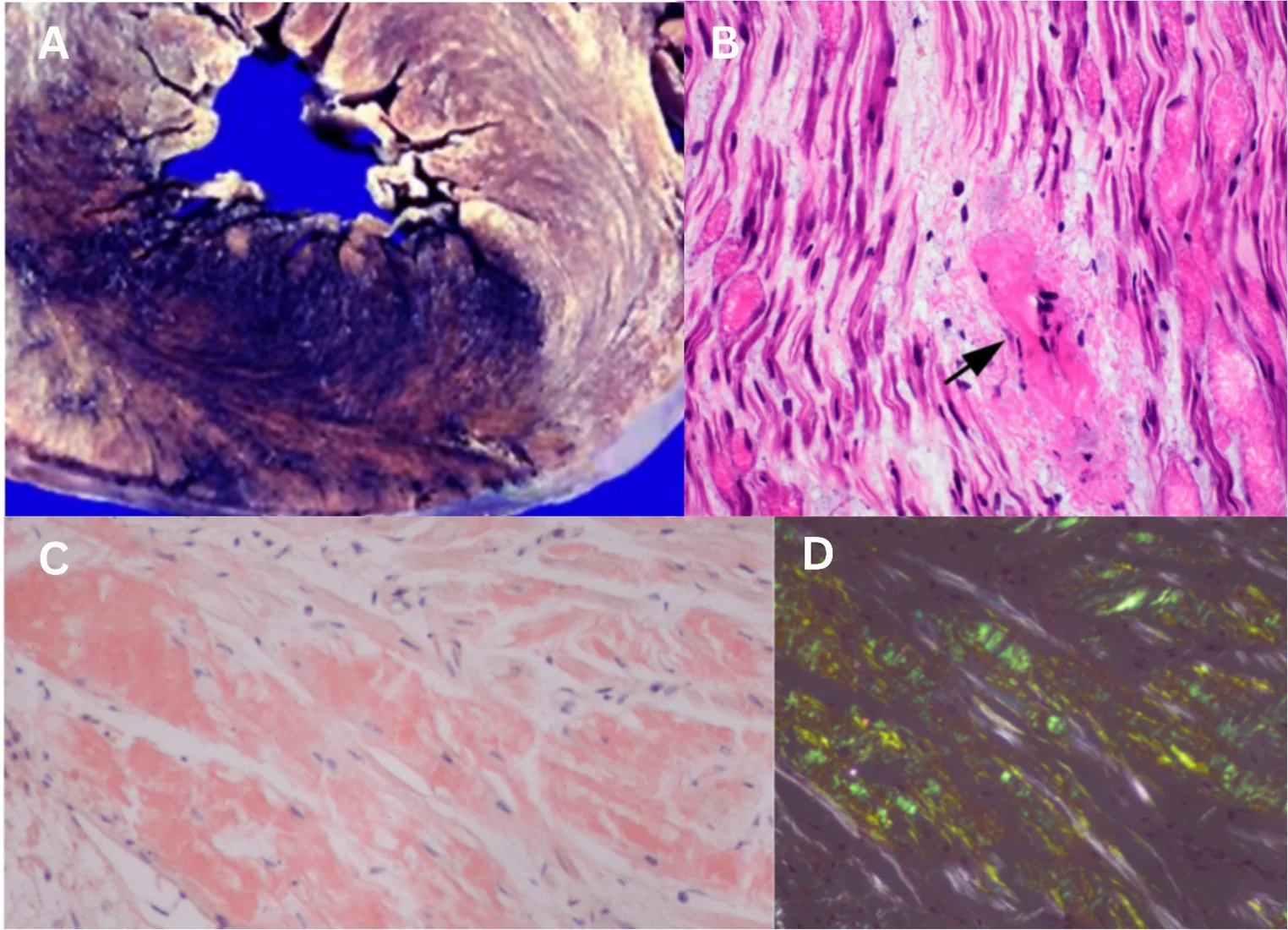
**Staining Methods for Amyloid. (A)** Lugol’s stain on myocardial section from a patient with cardiac amyloidosis. Lugol’s solution contains elemental iodine and potassium iodide, and tests for the presence of starch by coloring it blue. It is a derivative of the original iodine sulfuric acid test used by J.J. Colin and H.F. Gaultier de Claubry in 1814. Figure adapted from Wick [62] **(B)** Amyloid deposits in peripheral nerves stained with crystal violet. Like methyl violet, crystal violet is metachromatic and colors amyloid reddish purple. Figure adapted from Cai [63] **(C)** Congo red staining of amyloid in cardiac tissue from a patient diagnosed with senile systemic amyloidosis. Amyloid deposits are colored red. Figure adapted from Tanskanen [2] **(D)** Amyloid stained with Congo red turns apple-green in polarized light. This is due to birefringence exhibited by the dye molecules. Figure adapted from Tanskanen [2]

The first suspected identifications of amyloidosis were made in the abdominal organs and likely represented the secondary AA amyloidosis seen in diseases of chronic inflammation. In 1639, Nicolaus Fontanus noted that his patient’s spleen at autopsy was found to be pale, stiff, and waxy; while unconfirmed, this is potentially the earliest description of what is now known as the “sago spleen” found in secondary or AA amyloidosis [1]. This condition was also recorded by Thomas Bartholin in the first volume of his work *Historiarum Anatomicarum Rariorum* in 1654, in which he described the white deposits as hardening the spleen such that it was barely able to be dissected [1]. Almost a century later, several other researchers observed similar “clay-colored pituitous substances” in their patients’ livers and kidneys that hardened when exposed to heat, leading them to believe the material was albuminous in nature. Yet the term “amyloid” did not come into existence until another century later, coined in 1838 by Matthias Schleiden [2]. In addition to medicine and human anatomy, Schleiden studied botany and was interested in applying the iodine-sulfuric acid test created by J.J. Colin and H.F. Gaultier de Claubry to localize starch within a given plant cell [1–3]. Schleiden believed that the staining reaction showed the physical transformation of the plant cell material into starch and so he used the word “amyloid” to describe his findings, coming from the latin “*amylum*” meaning “starch” [2]. It was Rudolf Virchow who first used the term “amyloid” in a medicinal context to describe the small, circular spots found in the nervous system that presented with the same blue color as starch when given the iodine-sulfuric acid stain (Fig. 2) [2, 4]. Virchow labeled these small masses the “corpora amylacea,” a term which still persists today [2, 3].

In 1859, Carl Friedreich and August Kekulé concluded that amyloid deposits were not made of starch or cellulose, but rather of an albuminoid compound due to high nitrogen content in the tissue [1, 5]. Virchow challenged these findings along with similar prior claims by Schmidt and Budd, arguing that chemically analyzing an affected patient’s entire liver was a flawed methodology that could not accurately characterize the isolated disease-causing substance [1]. Johann Meckel agreed with Friedreich and Kekulé that amyloid was neither starch nor cellulose, but he incorrectly hypothesized that the iodine-reactive deposits were made of cholesterine, harkening back to the ‘lardaceous’ appearance of affected organs [1]. Despite these ongoing debates over its true chemical nature, Virchow’s immense influence in the field ensured that his misnomer, ‘amyloid,’ became a permanent fixture in medical vocabulary; modern science has since proven that none of these early researchers correctly identified the true composition of amyloid.

### Staining methods

It is indisputable that Virchow’s use of the iodine-sulfuric acid test for amyloid kickstarted the development of current methodologies for amyloid recognition, although he ironically opposed these new techniques. The accidental success of the iodine-sulfuric stain led researchers Victor Cornil, Richard Heschl, and Rudolf Jurgens to all individually discover the methyl violet stain in 1875. [1, 2] An aniline dye, the stain was reported to be “metachromatic” meaning the dye would change from purple to a reddish pink when bound to amyloid. ^1,2^ It is this stain that allowed Isidor Soyka to classify the first case of cardiac amyloidosis in 1876, an invaluable discovery for amyloidosis research [2]. His classification separated this form of amyloidosis from secondary (AA) amyloidosis, as he did not associate it with a chronic illness or infection [6]. Soyka is also credited with creating the term “senile amyloidosis” to describe his findings, as the amyloid buildup in his patient was caused by old age; a name used for decades to come [7].

Almost fifty years later, use of the Congo red dye in humans began serendipitously. Originally created as a color for textiles, its distinctive name may have been a marketing tactic during its introduction at the “Congo conference” in Berlin [8]. Another 40 years later, while attempting to measure blood volume and assess liver function, Hermann Bennhold made a crucial discovery: he found that Congo red left the bloodstream faster in patients with amyloidosis, allowing identification to be done in real-time on a living patient rather than on tissue sections during an autopsy [9]. The first description of the characteristic “apple-green” color emitted by amyloid deposits under polarized light is credited to Paul Ladewig in 1945, but the underlying phenomenon of birefringence after Congo Red staining was noted much earlier by Paul Divry and Marcel Florkin in 1927 [9].

## Amyloid protein structure

### Amyloid as an immunoglobulin

In the mid to late 19th century, cases reported by Hermann Weber (1867) and Carl Wild (1886) described patients with likely AL amyloidosis [1]. By the early 1900 s, the Bence Jones protein had been identified in patients with bone diseases like multiple myeloma and was suspected to be albumin-like, but it wasn’t until 1931 that Adolf Magnus-Levy suggested that the Bence Jones protein might be the substance responsible for amyloidosis. [1, 10] He believed the mechanism of action to be a combination of overproduction and decreased breakdown of the Bence Jones protein. 11 In the following decades, this philosophy grew, with researchers finding amyloid in the heart, lung, skin, vascular system, and tongue in patients with multiple myeloma., contributing to the current recognition of amyloid locations in primary (AL) amyloidosis and further differentiating it from those of secondary (AA) amyloidosis (liver, spleen, and kidneys) [1, 12]. Apitz also speculated that there was a compositional difference between the amyloid in secondary amyloidosis and the amyloid found with Bence Jones protein, which turned out to be correct [12]. This led to experiments studying the physical structure of the amyloid protein, using X-ray diffraction to identify its antiparallel β-pleated sheet pattern and sequence analysis to compare the amino acids of the amyloid protein and Bence Jones protein; they were both kappa-light chains [13, 14] From this, it was further proven in 1971 that amyloid proteins could be formed from the lambda-light chain portion of Bence Jones protein, solidifying the belief that the amyloid protein was indeed an immunoglobulin light chain [15].

## Serum amyloid A protein

Initially, it was assumed that all versions of the protein that caused the various amyloidosis manifestations were of the same light chain structure as that of AL amyloidosis. However, it was not long until the door was opened for the identification of other amyloid proteins, the earliest being serum amyloid protein A in 1972 [16, 17]. The recognition of secondary (AA) amyloidosis finally explained the earliest pathological descriptions of amyloid in patients with chronic systemic infection or inflammation, officially distinguishing it from AL amyloidosis While AA amyloidosis predominantly drives renal failure, its identification paved the way for discovering other precursor proteins with far greater cardiac penetrance.

## Familial amyloid polyneuropathy

The clinical diagnosis of Familial Amyloid Polyneuropathy (FAP) occurred before the pathologic understanding and causation of disease. In 1929, the first suspected case described a patient with prolonged pain and numbness in his extremities caused by an unknown substance found in his peripheral nerves; the patient had three siblings who passed away from the same condition [1]. Mario Corino de Andrade is the scientist traditionally referenced for the diagnosis of FAP, with his 1952 report consisting of over 70 patients from Portugal, dating as far back as 1939 [18]. Initially, FAP was regarded as an endemic condition due to its concentration within certain hotspots across the world (Portugal, Sweden, and Japan being the largest). However, across all locations, peripheral neuropathy caused by amyloid protein deposits was the glaring diagnosis, differing only in the age of onset [1, 18–20].

In 1978, Pedro Costa set out to uncover the protein responsible for FAP which he suspected to be different than either the previously described immunoglobulin or serum amyloid A proteins [21]. Upon separation and purification, Costa found that the amyloid fibril was indeed new and unique to FAP, although it did have the same β-pleated structure as all other amyloid proteins [21]. Furthermore, the protein was found to be almost identical to prealbumin with minor chemical differences, postulating that its pathogenicity resulted from a genetic mutation [21]. While early amyloidosis models relied on the overproduction of precursor proteins, subsequent sequencing of this prealbumin variant in the early 1980 s revealed a different mechanism. A dissimilarity in one to three amino acids between regular prealbumin and the pathogenic prealbumin suggested that it arose from a genetic mutation [21]. The name for this protein further developed into what we now know as transthyretin (TTR, although prealbumin is still a perfectly acceptable and synonymous term) due to the revelation of the protein’s binding affinities for thyroxine and retinol [22, 23].

### Understanding the transthyretin protein

The discovery of TTR as the precursor protein for FAP introduced a new segment of amyloidosis research, with scientists fervently trying to identify the causative mutation for the condition. In 1984, Maria Saraiva identified the mutation in Portuguese FAP as the now well-recognized Val30Met substitution, and this was similarly recognized by Satoru Tawara in Japan in 1983, making these the very first descriptions of a genetic cause for a hereditary form of amyloidosis [24, 25]. The use of polymerase chain reaction rapidly accelerated the process of discovering other TTR mutations, with hundreds being recognized today [2].

While Soyka is renowned for identifying amyloid within the heart via histology and pathology, the cause of the disease was still unknown at the time. In the following decades, post-mortem autopsies continued to locate amyloid deposits within the hearts of elderly individuals, more often in men than women, with most of the amyloid located in the atria, cardiac valves, vessels, and ventricular myocardium; this distinction changed the name of the condition to “senile cardiac amyloid” – a step above Soyka’s previous designation of just “senile amyloid”. This title was updated once again after it was noticed that the amyloid could extend beyond the cardiac tissue, thus becoming “senile systemic amyloidosis” [26].

Through several different publications in the 1980 s, Per Westermark characterized the amyloid protein as being derived from prealbumin rather than merely related to it [27]. He originally thought that senile systemic amyloidosis (SSA) was a fourth type of amyloidosis, separate from AL, AA, and FAP; however, his interest in understanding SSA led him to further uncover that the TTR found in SSA was considered “normal” prealbumin, unlike the mutated TTR that caused FAP [26, 27]. Therefore, as is now known, the proteins for FAP and SSA are not distinct but rather different versions of one another [26]. While this may seem simple and slightly redundant, this information provided the foundation for the current understanding of cardiac amyloidosis and the significance of genetic testing in the diagnostic process.

## Non-invasive diagnosis of amyloidosis

In the 1970 s, technetium-labeled nuclear radiotracers like pyrophosphate (PYP) were used to identify areas of myocardial tissue necrosis [28]. These bone-avid agents were postulated to bind to calcified tissue in the peripheral zones of infarcted tissue and were imaged using scintillation cameras [29]. Some early studies with PYP used pathologic correlation to identify this necrotic tissue, though some cases had persistently positive PYP scans and histopathology revealed amyloid deposits. Alongside this growing clinical recognition of cardiac amyloidosis, in 1982 there were three case series noting cardiac uptake of PYP on planar scintigrams in patients with biopsy proven extra-cardiac amyloidosis [30–32]. At the time, it was postulated that these bone-avid agents bound to the calcium-dependent serum amyloid P component adjacent to and connecting amyloid fibrils; this was later disproven and the modern theory is that these tracers bind to microcalcifications (more prevalent in ATTR than in AL) embedded around amyloid fibrils [33]. Theodore Wizenberg proposed grading the uptake as 0 (normal scan), 1+ (faint activity, likely blood pool), 2+ (definite activity in the myocardium but less intense than adjacent normal bone), 3+ (activity in the myocardium equal to bone), and 4+ (activity in the myocardium greater than bone) [30]. The use of these bone-avid tracers in amyloidosis did not initially gain traction due to a series of negative studies in patients with AL amyloidosis. Enrica Perugini noted that technetium-99 m DPD (3,3-diphosphono-1,2-propanodicarboxylic acid) was taken up only in the hearts of patients with ATTR and not AL amyloidosis [34]. As such, Perugini essentially condensed grades 1 and 2 into a single category where planar uptake in the cardiac silhouette appeared less than bone. Thus, the eponymous Perugini 0 to 3 grading system was born and is still used today [28, 35, 36].

## The modern understanding of amyloid subtypes

### Transthyretin amyloidosis

The TTR protein in its native state is a 127 amino acid homotetramer; the adverse symptoms of amyloidosis are caused when these subunits dissociate, resulting in monomeric proteins that misfold and improperly aggregate into tight, insoluble, amyloid fibrils [22, 23] In wild-type amyloidosis, or ATTRwt, this dissociation is spontaneous and most commonly attributed to the weakening of protein-folding mechanisms with age [22, 23]. The partial or complete unfolding of ATTR can also be caused by biochemical or physical factors, such as enzymatic cleavages and/or mechanical stress [37].

ATTR definitively causes cardiomyopathy, in both the wild-type and hereditary types, although ATTRwt can only result in cardiomyopathy whereas variant ATTR exhibits both cardiomyopathy and polyneuropathy [38]. This cardiomyopathy is achieved by the deposition of amyloid fibrils between myocardial cells, decreasing pumping capacity for both systolic and diastolic function and resulting in heart failure with preserved ejection fraction (HFpEF) [38, 39]. While recognition is much more prevalent these days, ATTR amyloidosis can still go undetected for many years because it is extremely slow-developing and almost always presents with other conditions common in elderly populations (i.e. hypertension and cardiac arrhythmia) [39]. Additionally, cardiac amyloidosis often causes hypertrophy of the left ventricle, which can distort detection and further delay treatment [39]. The assumption that intervention for amyloidosis may be futile for older patients may also contribute to the lack of appropriately diagnosed cases [40].

The other type of ATTR amyloidosis, known as hereditary or variant ATTR (ATTRv), results from a missense mutation in the TTR gene that produces slight conformational changes in the TTR protein. ATTRv is the agent of development for FAP, with over 100 different mutations recognized [41]. Some of these mutations are localized to specific regions of the world and regarded as “founder mutations” due to the high prevalence in their country of origin; examples include the p.Thr80Ala (T60A) variant in northwest Ireland, p.Val50Met (V30M) in Portugal (also Sweden and Japan), p.Val142Ile (V122I) in African countries, and p.Ala117Ser (A97S) in China and Taiwan [42]. Transmitted through an autosomal dominant pattern, all TTR mutations force the dissociation of its tetramer structure by destabilizing the interactions between subunits; for example, the V30M mutation is thermodynamically destabilizing, whereas the V122I mutation is kinetically destabilizing [41, 42].

Each genetic mutation in ATTRv can cause a slightly different phenotype, and this can vary even more between patients and even within families. ATTRv has been noted to exhibit some incomplete penetrance despite possessing an autosomal dominant pattern of inheritance, further diversifying numerous mutations, phenotypes, and penetrance rates within a given population [42]. Generally, the variants exist on a spectrum of being more likely to cause polyneuropathy, autonomic neuropathy, or cardiomyopathy. The V122I variant is the most commonly recognized mutation for ATTRv (although it is extremely rare outside of the African American population- its traditional ethnic group), and its cardiac involvement is significantly more severe than that of ATTRwt with an earlier onset of disease and more rapid progression [38, 39]. The V30M mutation is known to affect the peripheral nerves much earlier than it does the heart, so by the time a cardiac diagnosis is made, patients already have severe peripheral and/or autonomic neuropathy; V30M is further divided into early and late onset variants [38, 41]. Because of the wide variety in symptom presentation, it is vital to perform genetic testing during a diagnosis of ATTR amyloidosis to ensure the proper treatment is administered.

As TTR is synthesized in the liver, liver transplantation used to be the only effective treatment for amyloidosis by removing the source of the disease, with the first one being performed in 1990 in Sweden [43, 44]. However, in modern medicine, currently approved therapies for ATTR amyloidosis aim to stop the proliferation of additional amyloid deposits by limiting the number of misfolded TTR proteins. Gene silencers harness the RNA interference mechanism within hepatocytes in order to destroy messenger RNA, yielding a dramatic reduction in TTR protein levels, whereas protein stabilizers bind to the tetrameric TTR protein to reduce protein misfolding [22, 39, 45]. Drugs currently under development include gene editing using the CRISPR-Cas9 system which would also reduce further amyloid deposits, as well as monoclonal antibodies aimed at depleting/removing amyloid fibrils deposited in tissue [22, 39, 45].

### Light chain amyloidosis

Formerly called primary amyloidosis, AL amyloidosis is a monoclonal plasma cell disorder caused by the excessive production and incorrect folding of immunoglobulin light chains, leading to amyloid fibril buildup in multiple organs including the heart, kidneys, liver, GI tract, and nervous system [39, 46]. The disease is sometimes accompanied by multiple myeloma if CRAB criteria are present or bone marrow involvement is significant, as they both originate from the disordered proliferation of plasma cells in the bone marrow [47]. The immunoglobulin light chains can be either kappa or lambda variations, and measuring the free light chain levels is used as both a diagnostic tool and a biomarker to track progression of the disease and response to treatment [47]. However, AL amyloidosis is often a multi-organ system disease and can be difficult to detect before the amyloid has involved many organs [47]. The current treatment regimen of choice for AL amyloidosis is a combination of daratumumab, cyclophosphamide, bortezomib, and dexamethasone (dara-CyBorD) based on the ANDROMEDA study [48, 49]. In those with t(11;14) cytogenetics on the bone marrow, venetoclax is the first option for second-line therapy [49]. Bone marrow transplantation is being used less frequently at this point due to the high efficacy and low toxicity of the aforementioned treatments. Several amyloid-depleting drugs have been studied for AL amyloidosis, though none have shown consistent results in affecting patient outcomes [50].

### Other types of amyloidosis

AA amyloidosis results from the systemic deposition of serum amyloid A (SAA) protein, an acute-phase reactant produced by the liver in response to chronic inflammatory stimuli [51]. This condition is typically secondary to long-standing inflammatory diseases such as rheumatoid arthritis, inflammatory bowel disease, or chronic infections like tuberculosis and osteomyelitis [51, 52]. The kidneys are the primary organs involved, often presenting as nephrotic-range proteinuria and progressing to end-stage renal disease; however, the liver and spleen are also frequent sites of deposition [51, 52]. AA amyloidosis must be diagnosed via tissue biopsy, often from the kidney or a surrogate site like the abdominal fat pad [51, 52] Current treatment strategies focus almost exclusively on aggressive control of the underlying inflammatory driver to lower SAA levels below 10 mg/L [51]. This includes the use of biologic agents such as TNF-alpha inhibitors or IL-6 receptor antagonists (e.g., tocilizumab), which have shown efficacy in stabilizing or even reversing renal dysfunction [51].

**Table 1:** **Categories of Amyloid Fibril Proteins.** All 42 amyloid fibril proteins recognized by the International Society of Amyloidosis (ISA) as of 2024 [61]. Amyloid-causing proteins are categorized as genetic variant (“Hereditary”), non-mutated wild type (“Acquired”), or both. Bolded proteins are capable of causing systemic amyloidosis

**Hereditary**	**Acquired**	**Hereditary or Acquired**
AApoA-I	**AH**	**AL**
AApoA-II	**ALECT2**	**AA**
AApoC-II	ATMEM106B	**ATTR**
AApoC-III	**ACal**	**Aβ2M**
AGel	AIAPP	**AApoA-IV**
ALys	AANP	Aβ
AFib	APro	AaSyn
ACys	ASom	ATau
ABri	AGluc	**APrP**
ADan	APTH	
	AIns	
	AEnf	
	AGLP1	
	**AIL1RAP**	
	ASPC	
	ACor	
	AMed	
	AKer	
	ALac	
	AOAAP	
	ASem1	
	ACatK	
	AEFEMP1	

**Fig. 3:**
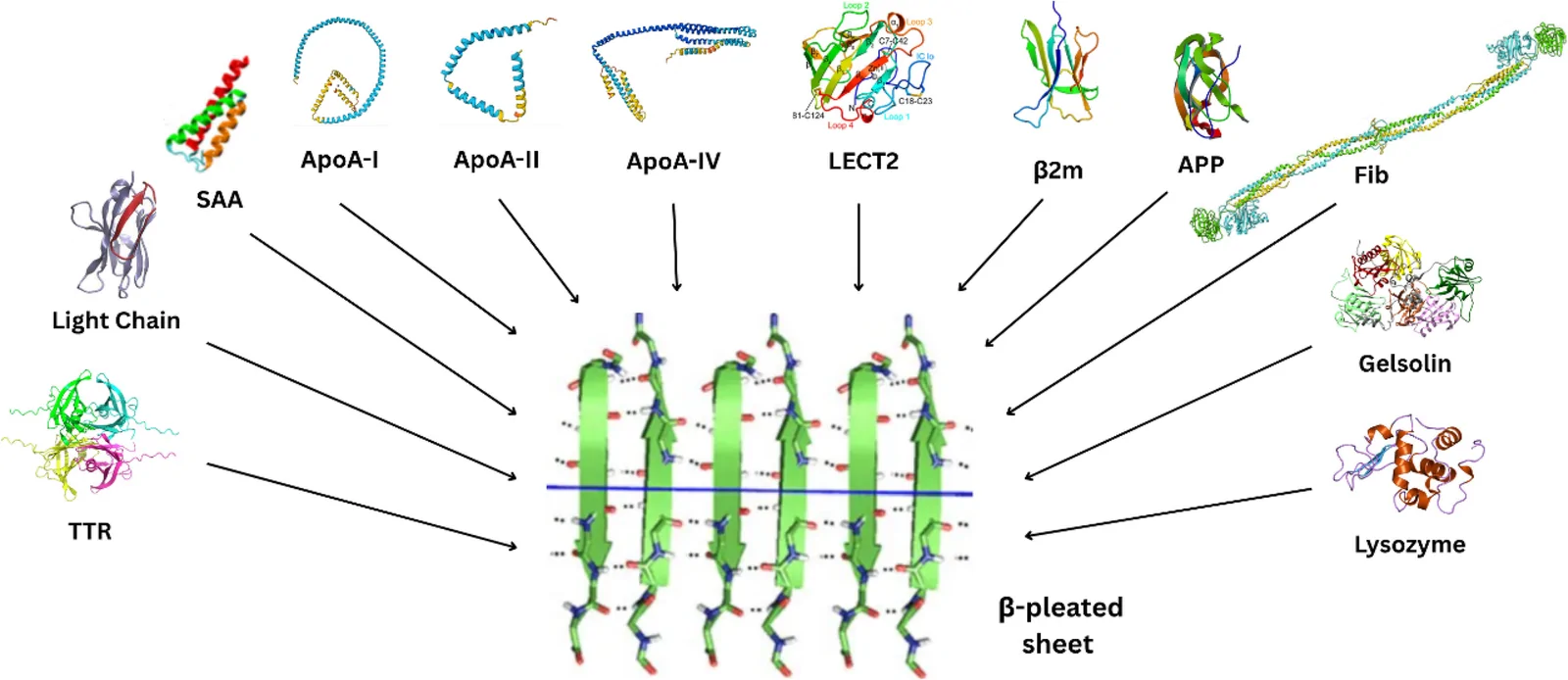
Precursor Proteins for Types of Amyloidosis. Several of the protein monomers for the various kinds of amyloidosis mentioned in this paper are shown. Regardless of disease type, each protein ultimately aggregates into a β-pleated sheet structure [64], with some differences noted among precursor proteins. Common proteins depicted include transthyretin (TTR) [65], light chain [66], Serum Amyloid A (SAA) [67], Apolipoprotein A-I (ApoA-I) [68], Apolipoprotein A-II (ApoA-II) [68], Apolipoprotein A-IV (ApoA-IV) [68], Leukocyte cell-derived chemotaxin 2 (LECT2) [69], ß2-microglobulin (β2m) [70], amyloid-ß precursor protein (APP), Fibrinogen Aα-chain (AFib) [71], gelsolin, and lysozyme

Several rarer forms of systemic amyloidosis are increasingly recognized due to improvements in proteomic typing (Table 2; Fig. 3). ALECT2 amyloidosis, involving leukocyte chemotactic factor 2, is now noted as a significant cause of renal amyloid in patients of Hispanic, Native American, and Middle Eastern descent; unlike other forms, it lacks a known hereditary link or effective targeted therapy [53, 54]. Other rare variants include hereditary fibrinogen Aalpha-chain (AFib), which primarily targets the kidneys, and apolipoprotein (AApoA-I, II, and IV) amyloidoses, which can involve the heart and liver [54–56] ß2-microglobulin amyloidosis (Aß2), historically associated with long-term hemodialysis, has seen a decline in prevalence due to modern high-flux dialysis membranes [57].

**Table 2:** **Most Common Types of Systemic Amyloidosis**. The four most prevalent subtypes of amyloidosis are listed along with their defining characteristics. ATTR, AL, and AA make up the majority of diagnosed cases of amyloidosis, although their symptomatic presentation and respective treatments differ greatly from one another. CM = cardiomyopathy, PN = polyneuropathy

Type	Precursor Protein	Major Organ Involvement	Most Common Diagnostic Method	Treatment
ATTRwt	Normal transthyretin	Cardiac, tendons/ligaments	Cardiac amyloid radionuclide imaging AL rule out	Tafamidis (CM only) Acoramidis (CM only) Vutrisiran
ATTRv	Mutated transthyretin	Cardiac, Nervous system	Cardiac amyloid radionuclide imaging AL rule out Genetic testing	Tafamidis (CM only) Acoramidis (CM only) Vutrisiran Eplontersen (PN only)
AL	Immunoglobulin light chain (kappa or lambda)	Multisystem	Free kappa/lambda light chains Serum/urine immunofixation Tissue biopsy Bone marrow biopsy	Dara-CyBorD
AA	Serum amyloid A	Multisystem	Tissue biopsy	TNF-alpha inhibitors IL-6 receptor antagonists

### Future directions

Treatment options for amyloidosis continue to be investigated (Table 2), with gene editing and monoclonal antibodies emerging as the leading two approaches. Utilizing the CRISPER-Cas9 system, gene editing inactivates the gene responsible for synthesizing the TTR protein, achieving the same goal as RNA silencing therapies [22, 45]. MAGNITUDE, a phase-3 clinical trial for this therapy, is currently underway and is showing promising results of up to 95% reduction in TTR levels [22]. Monoclonal antibodies introduce a new strategy to amyloidosis management, using immunotherapy to remove already-deposited amyloid fibrils from affected organs [39, 45]. Known as TTR depleters, the antibodies are designed to specifically target misfolded TTR and are the first amyloidosis therapy attempting to eliminate aggregates without affecting normal TTR production, extending beyond merely halting disease progression [39, 45 Several clinical trials are ongoing, including the phase 3 DepleTTR-CM and CLEOPATTRA trials [45]. Unfortunately, several depleter programs for AL amyloidosis have been shut down due to negative trials.

Just as treatments are being improved, so too are detection methods for amyloidosis. While PYP scans are the primary imaging modality to accurately diagnose ATTR amyloidosis, novel positron emission tomography radiotracers are being studied [58].There are three phase 3 trials underway, PETAL, CARdiag, and REVEAL, that are investigating the accuracy of various tracers [58]. Detection using artificial intelligence has also increased in the recent years, with several companies pioneering deep learning algorithms based on echocardiograms to improve the detection of this rare disease [59, 60]. All of these technologies and imaging techniques eliminate variability in human interpretation, leading to potentially earlier and more accurate diagnoses of amyloidosis.

## Conclusion

The history of amyloidosis is a testament to the cumulative nature of medical discovery. What began as vague descriptions of “sago” and “lardaceous” organs in the 17th and 18th centuries eventually yielded to the histological precision of Congo red birefringence. The persistent curiosity of early researchers ultimately dismantled the “starch” hypothesis, leading to the critical understanding that amyloidosis is not a single entity, but a diverse group of protein misfolding disorders. Whether driven by plasma cell dyscrasias, chronic inflammation, or genetic mutations, the subsequent identification of specific precursor proteins has fundamentally shifted clinical management. Today, the historical reliance on post-mortem diagnosis has been replaced by non-invasive nuclear imaging, and the era of strictly supportive care has given way to disease-modifying therapies, including gene silencers and targeted drug regimens. While Virchow’s misleading botanical name persists, the future of amyloidosis care is firmly rooted in advanced molecular medicine.

## Data Availability

No datasets were generated or analysed during the current study.
